# A new species of *Pupulina* van Beneden, 1892 (Copepoda, Siphonostomatoida, Caligidae) from Aetobatuscf.narinari (Pisces, Myliobatidae) from the Pacific coast of Ecuador

**DOI:** 10.3897/zookeys.777.26017

**Published:** 2018-07-30

**Authors:** Yanis Cruz-Quintana, Víctor Caña-Bozada, Eduardo Suárez-Morales, Ana María Santana-Piñeros

**Affiliations:** 1 Departamento Central de Investigación, Universidad Laica Eloy Alfaro de Manabí, Ciudadela Universitaria, Calle 12, Vía San Mateo, Manta EC130802, Manabí, Ecuador Universidad Laica Eloy Alfaro de Manabí Manabí Ecuador; 2 Grupo de Investigación en Sanidad Acuícola, Escuela de Acuicultura y Pesquería, Facultad de Ciencias Veterinarias, Universidad Técnica de Manabí, Ciudadela Universitaria, Leónidas Plaza EC131402, Bahía de Caráquez, Manabí, Ecuador Universidad Técnica de Manabí Manabí Ecuador; 3 El Colegio de la Frontera Sur (ECOSUR) Unidad Chetumal. Av. Centenario Km. 5.5, Chetumal, Quintana Roo 77014, México El Colegio de la Frontera Sur Chetumal Mexico

**Keywords:** crustaceans, Eagle ray, parasites, taxonomy

## Abstract

A new caligid copepod species, *Pupulinamantensis***sp. n.** is described based on female and male specimens collected from the gills of the myliobatid elasmobranch Aetobatuscf.narinari Euphrasen, 1790 captured off the Pacific coast of Ecuador. The new species has a unique combination of characters that diverges from its known congeners, including: (i) weakly developed posterolateral processes on the genital complex; (ii) large spines on posterior surface of maxilliped basis (iii) abdomen slender, unsegmented, approximately 1/2 length and 1/5 width of genital complex; (iv) third exopodal segment of leg II with single long naked spine adjacent to minute, naked lateral spine; (v) velum of leg II with adjacent patch of denticles; (vi) caudal rami slightly less than half the length of genital complex; (vii) post-antennal process with robust, posteriorly directed tine, sclerotized stump posterolaterally, and two multi-sensillate papillae located on or near base of process (viii) post-oral process oval. The overall prevalence of *P.mantensis***sp. n.** on its host was 37.5% and its mean abundance was 1.87 specimens per host. This is the second record of the genus *Pupulina* from Ecuador and the second record of *Pupulina* infecting rays of the Myliobatinae genus *Aetobatus*, of the subfamily Myliobatinae, after its discovery on *A.ocellatus* in Australia, thus confirming this expansion of its previously known host range to a new elasmobranch subfamily.

## Introduction

Species of *Pupulina* Van Beneden, 1892 have been reported from rays of the genera *Mobula* Rafinesque, 1810 and *Manta* Bancroft, 1829 from eastern Pacific, the Gulf of Mexico and South Africa (Wilson 1952, Dippenaar and Lebepe 2013). Members of this caligid genus appear to be restricted to species of the ray family Mobulidae (Dojiri and Ho 2013). *Pupulina* is distinguished from other caligid genera by its possession of: (1) posterolateral processes on the female genital complex , (2) a small conical processes posteromedial to dentiform maxillulary projection, (3) a dentiform or membranous process immediately posterior to the maxilliped, (4) a well-developed endopod of leg I, (5) inflated outer margin of the first and second endopodal segments of leg II and first endopodal segment of leg III, (6) distinctly 3-segmented rami of leg III, and (7) the armature of the exopod of leg III (Wilson 1952, Dojiri and Ho 2013) (8) a 2-segmented endopod of leg I armed with 0-0; 3 setae. The genus includes six valid species (Walter and Boxshall 2016): *Pupulinaflores* van Beneden, 1892 from *Mantabirostris* Walbaum, 1792 in Azores and Galápagos Islands; *P.brevicauda* Wilson, 1952 and *P.minor* Wilson, 1952 from *Mobulalucasana* Beebe & Tee-Van, 1938 in Santa Catalina, California and *M.diabolus* Shaw, 1804 in Trivandrum, India; *P.cliffi* Dippenaar & Lebepe, 2013 from *Mobulakuhlii* Müller & Henle, 1841 and *M.eregoodootenkee* Bleeker, 1859 in off Umdloti, South Africa; *P.merira* Dippenaar & Lebepe, 2013 from *Mobulakuhlii* and *M.eregoodootenkee* from off Karridene, South Africa, and *P.keiri* Boxshall, 2018 from *Aetobatusocellatus* Kuhl, 1823 from Moreton Bay, Australia.

The white-spotted eagle ray, *Aetobatusnarinari* (Euphrasen, 1790) inhabits inshore areas and coral reef environments (Schluessel et al. 2010). *Aetobatusnarinari* is found circumglobally throughout temperate and tropical waters; however, some studies have suggested that this nominal species may represent a species complex containing two or three species (White et al. 2010). As part of a research program on the helminth community parasitizing commercial fish of the Manabí coast, Ecuador, we found adult specimens of *Pupulina* that present an undescribed species. Based on male and female individuals, we herein describe the new species and compare it with its known congeners; and provide new data about the elasmobranch host range of the genus among the elasmobranchs.

## Materials and methods

**Figure 1.**
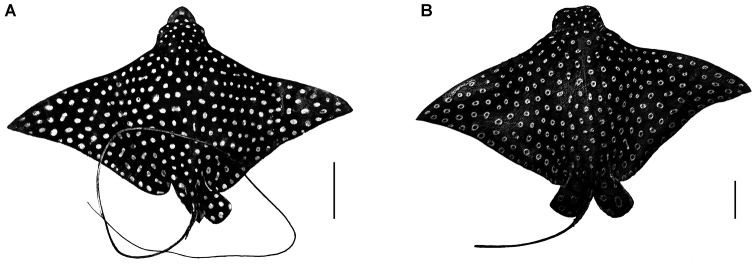
Aetobatuscf.narinari captured in Los Esteros, Manabí, Ecuador. **A** Morphotype 1, showing solid white spots **B** Morphotype 2, showing ocellated white spots. Scale bars: 10 cm.

Eight white-spotted eagle rays Aetobatuscf.narinari were captured and examined between February and June 2015 from Los Esteros beach (0°56'51"S – 80°41'44"W), State of Manabí, on the Pacific Coast of Ecuador. The rays are incidentally caught by artisanal fishermen during trawling in shallow water, but are discarded because they have no commercial value. However, some rays die during the trawling and are processed for research purposes. The rays were transported to the laboratory of Parasitology at the Universidad Laica Eloy Alfaro de Manabí (ULEAM) and digital photographs of the specimens were immediately obtained. Pictures of the dorsal spot pattern of each morphotype were prepared (Fig. 1A–B) for future identification of the individuals when the presumed species complex of *A.narinari* is solved (methodology followed Marie and Justine 2005). Copepods were obtained from the hosts by removing them with needles and then fixed in 70% ethanol for long-term preservation; they were cleared in gradually increasing concentrations of glycerol and mounted on slides sealed with glycerin jelly.

Drawings were prepared using a camera lucida attached to a CX 31 Olympus compound microscope at the laboratory of Universidad Técnica de Manabí. Unless otherwise stated, measurements are presented in micrometers (µm), and include the range and mean value in parentheses. Morphological terminology follows Boxshall et al. (2002). The ecological terms prevalence, mean abundance, and intensity were determined and used following Bush et al. (1997). Type specimens were deposited in the Zoology Museum of the QCAZ (QCAZ), Quito, Ecuador and the collection of Zooplankton held at El Colegio de la Frontera Sur (ECOSUR), Unidad Chetumal (ECO-CHZ), Quintana Roo, Mexico.

Two adult specimens, one female and one male, were prepared for SEM examination with a TOPCON SM-510 microscope at facilities of ECOSUR in Tapachula, Mexico. The preparation process included dehydration of specimens in progressively higher ethanol solutions (60–100%), critical point drying, and gold-palladium coating (20 nm) following standard methods.

## Results

Eight white-spotted eagle rays A.cf.narinari between 94 and 157 cm total length, were examined, all specimens showed a homogeneous spotted pattern on black disc, with relatively homogeneous spot size except on the head where spots are relatively smaller. Six of them displayed fully white spots (morphotype 1) (Fig. 1A), while the remaining two individuals displayed ocellate white spots (morphotype 2) (Fig. 1B).

### Order Siphonostomatoida Thorell, 1859

#### Family Caligidae Burmeister, 1835

##### Genus *Pupulina* van Beneden, 1892

###### Pupulina mantensis sp. n.

Taxon classificationAnimaliaSiphonostomatoidaCaligidae

http://zoobank.org/446CA43A-A40F-4612-A5D1-40014FC25A8C

####### Type host.

White-spotted eagle ray Aetobatuscf.narinari (Myliobatiformes, Myliobatidae).

####### Type locality.

Los Esteros beach (0°56'51.43"S – 80°41'44.90"O), Manta city, State of Manabí, Pacific coast of Ecuador. The specimens of A.cf.narinari were incidentally caught by local fishermen by trawling in shallow water (depth < 5 m).

####### Site on host.

Ventral body surface and gill filaments.

####### Prevalence.

Overall prevalence 37.5% (n = 8). Prevalence on morphotype 1, 50% (n = 6). Prevalence on morphotype 2, 0% (n = 2).

####### Mean abundance.

1.87 parasites per ray (n = 8). 2.5 parasites per morphotype 1 (n = 6).

####### Mean intensity.

5 parasites per infected ray (n = 3).

####### Type material.

Holotype adult female, undissected specimen preserved in 70%, ethanol vial (QCAZ No.3452); allotype adult male, undissected specimen preserved in 70% ethanol, vial (QCAZ No.3450); paratype adult male, undissected, preserved in 70% ethanol, vial (QCAZ No.3451); two paratype adult females, partially dissected, semi-permanent slides mounted in glycerin, sealed with Entellan (CO-CH-Z-10036); two paratype adult males, partially dissected, semi-permanent slides mounted in glycerin, sealed with Entellan (CO-CH-Z-10037).

####### Etymology.

The species name is a toponym; it refers to the type locality where it was collected, Manta City, off the Ecuadorian Pacific coast.

####### Diagnosis.

**Figure 2.**
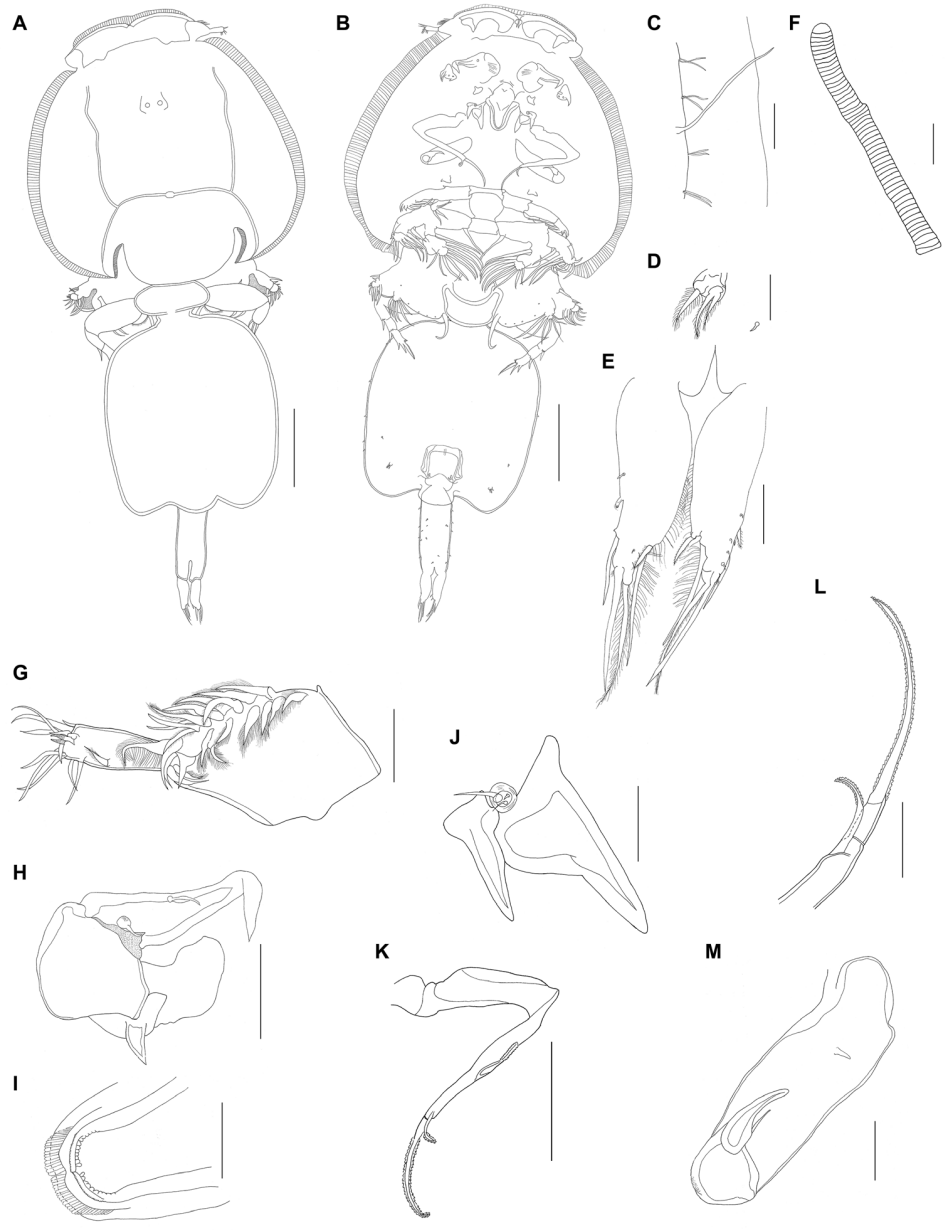
*Pupulinamantensis* sp. n., adult female. **A** habitus, dorsal view **B** same, ventral view **C** lateral border of cephalothorax, ventral view **D** leg V **E** caudal ramus **F** egg sac **G** antennule **H** antenna and post-antennal process **I** mandible **J** dentiform process of maxillule and post-oral process **K** maxilla **L** distal half of maxilla **M** maxilliped. Scale bars: **A, B** 800 µm; **D** 50 µm; **F** 450 µm; **H** 200 µm; **K** 400 µm; **C, E, G, I, J, L, M** 100 µm.

**Figure 3.**
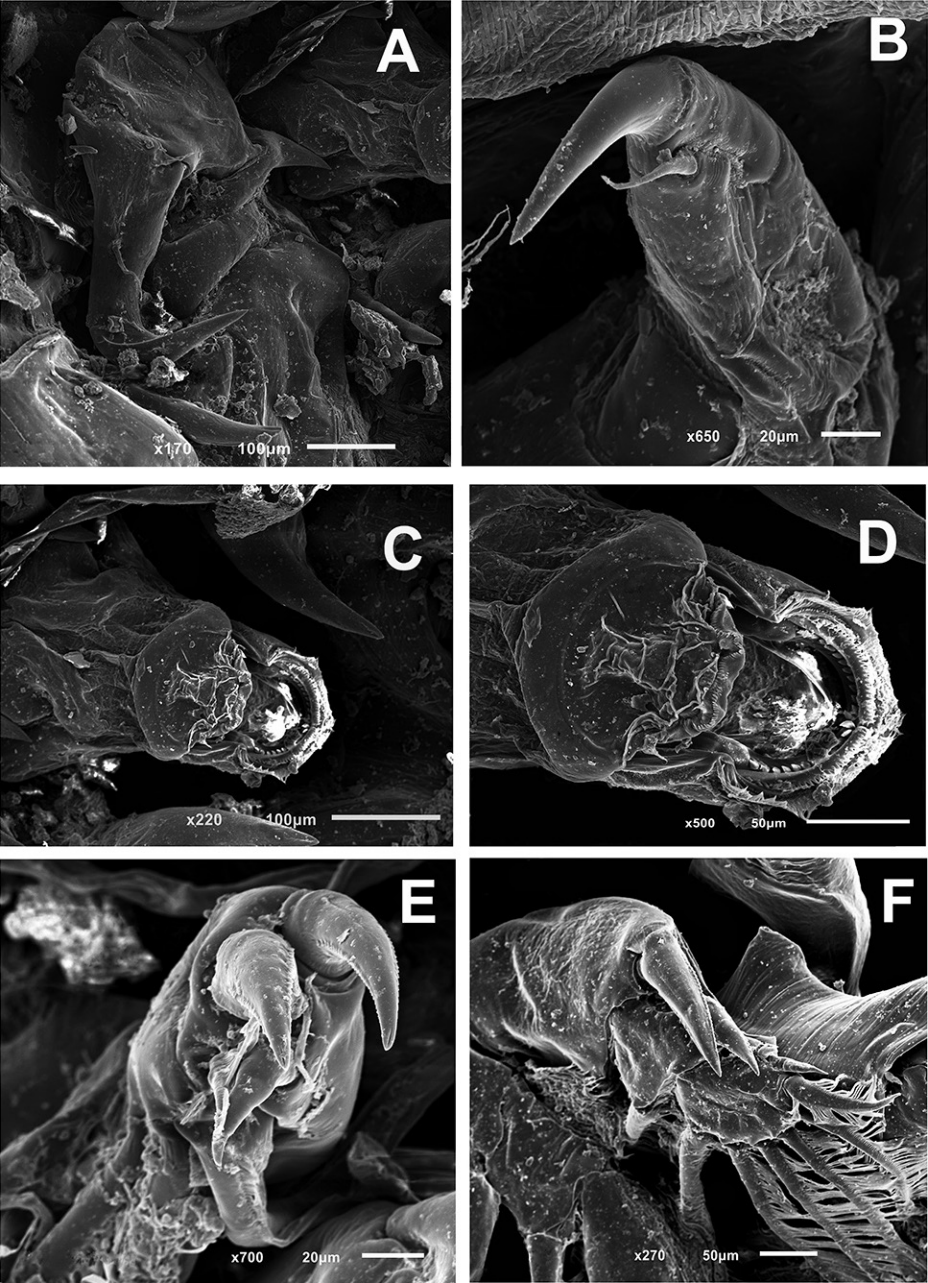
*Pupulinamantensis* sp. n., adult female. **A** antenna and post-antennal process **B** maxilliped **C** mouth tube **D** mandible **E** leg I **F** leg II.

**Figure 4.**
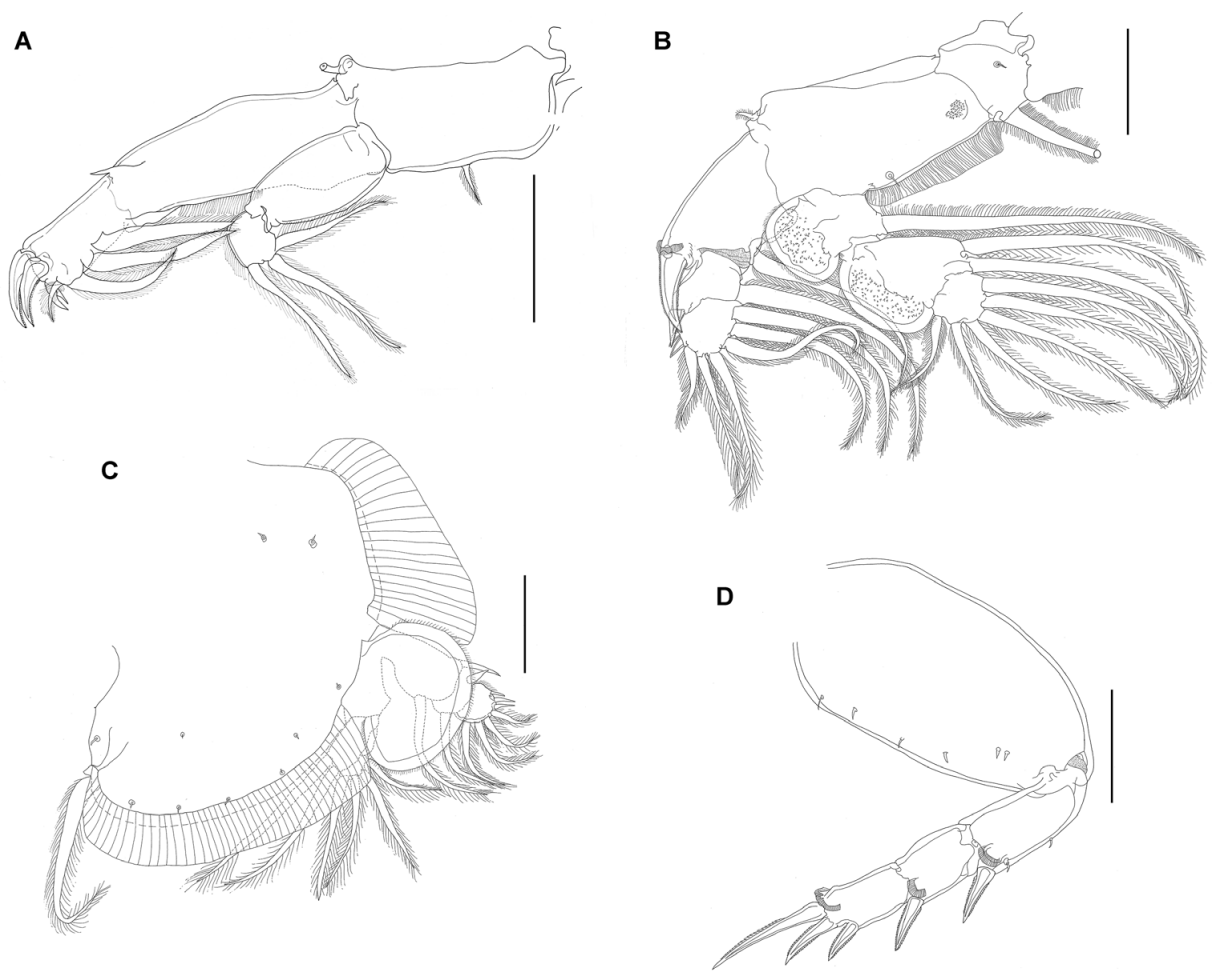
*Pupulinamantensis* sp. n., adult female. **A** leg I **B** leg II **C** leg III **D** leg IV. Scale bars: 200 µm.

The new species shows a unique combination of characters including (i) slight posterolateral processes on the genital complex (Fig. 2A, B); (ii) large cuticular spines located posterior to base of maxilliped (Fig. 2B); (iii) abdomen nearly 1/2 length and 1/5 width of genital complex (Fig. 2A, B); (iv) third exopodal segment of leg II with a single longer naked spine followed by a minute, naked spine on lateral margin (Fig. 4B); (v) velum of leg II bearing patch of denticles (Fig. 4B); (vi) caudal rami slightly shorter than half length of the genital complex (Fig. 2A, B); and (vii) post-antennal process with posteriorly directed robust tine, sclerotized stump posterolaterally, and two multi-sensillate papillae located on or near base of process (Figs 2H, 3A).

####### Description

(Figs 2–4). *Adult female.* Overall length, from anterior margin of frontal plate to distal margin of caudal rami (excluding egg sacs), 4.2–6.1 mm (5.4 mm; n = 6). Cephalothorax (2.7 mm × 2.8 mm) composed of cephalosome and first three thoracic somites (Fig. 2A–B). Carapace almost circular, slightly longer than wide, with obvious paired frontal plates lacking lunules, with shallow posterior sinuses; posterior margin convex (Fig. 2A). Anterior and lateral margins, as well as posterior sinuses of carapace, rimmed with hyaline, striated membrane (Fig. 2A). One large setule in middle portion of each side and pairs of short setules present at regular intervals along lateral margins (Fig. 2C). Antennules visible posterolaterally to frontal plate. Fourth free thoracic somite approximately twice as wide as long. Genital complex (Fig. 2A) nearly 3/4 length and 2/3 width of cephalothorax, rounded, with posterolateral corners forming slightly protruded processes; ventral surface with irregular pattern of small spinules and vestigial legs V posterolaterally (Fig. 2D). Spermatophores elongate, attached posteromedially on genital complex. Abdomen (Fig. 2A, B) indistinctly 3-segmented, almost 1/2 length and 1/5 width of genital complex; ventral surface with irregular pattern of small spinules. Caudal ramus (Fig. 2E) slender, around 1/2 length of abdomen, narrowed at apex, covered with spinules and ornamented with fringe of setules on inner margin. Armed with one pinnate outer seta nearly 2/3 from base, one naked seta outer distolaterally, one naked shorter and one large pinnate setae inner distolaterally, one pinnate and one naked setae distomedially approximately 2/3 length of caudal ramus. Egg sacs (Fig. 2F) uniseriate, each 2.9 mm long.

Antennule (Fig. 2G) 2-segmented. First segment armed with a double row of 22 stout pinnate setae inserted on anterodistal surface margin; apical segment with single pinnate seta posteromedially plus ten naked setae and three aesthetascs around apex. Antenna (Figs 2H, 3A) 3-segmented. First segment with large, posteriorly directed tine-like process; second segment subrectangular, unarmed; third segment forming curved claw, ornamented with membranous flap near distal hook, segment armed with short basal seta in basal region plus slender, naked setae inserted medially. Postantennal process (Figs 2B, 3A) weakly curved, with posteriorly directed robust tine, sclerotized stump posterolaterally, and two multi-sensillate papillae located on or near base of process. Mouth tube with intrabuccal stylet and strigil (Fig. 3C); mandible (Figs 2I, 3D) comprising four sections, bearing 12 apical teeth on inner margin. Maxillule (Fig. 2J) consisting of palp with one long and two shorter naked setae and large, subtriangular dentiform process. Sclerotized plate lateral to base of palp with a robust dentiform process directed posteriorly. Post-oral process present, consisting of raised, crescent-shaped sclerite located posteriorly to dentiform process. Maxilla (Fig. 2K) brachiform; basis with flabellum at approximately mid-length and distally with calamus and canna. Calamus nearly three times as long as canna, each rimmed with serrated membrane (Fig. 2L). Maxilliped (Figs 2M, 3B) with slender corpus, ornamented with medioventral spine; subchela (claw) slender, weakly curved, around half length of corpus, armed with relatively long naked seta on proximal 1/3. Post-maxillipedal process (Fig. 2B) present, consisting of pair of cuticular spines.

Leg I (Figs 3E, 4A) conspicuously biramous; sympod with one outer and one inner pinnate setae. Exopod 2-segmented; first segment armed with small outer distal spine and ornamented with usual row of setules along inner margin; second segment with pinnate apical seta IV much shorter than outermost spine I. Spines I and II bilaterally serrate; spines III shorter, not serrate, with well-developed accessory process. Inner margin with three large plumose setae. Endopod 2-segmented, almost same length as first exopod segment; first endopodal segment medially expanded, robust, unarmed; second segment with three inner pinnate setae and setules around lateral and distolateral margins.

Leg II (Figs 3F, 4B) biramous; sympod armed with short outer pinnate seta distally, plus two small outer setules on proximal position, one naked inner seta distally and long inner pinnate seta proximally; with small patch of spinules on outer proximal surface and inner margin fringed with narrow membrane. Exopod 3-segmented; first segment with setules on medial margin, long, pinnate seta distomedially and long, stout, bilaterally serrate distolateral spine, with pectinate membrane at base; Second segment bearing shorter, serrate spine distolaterally and long, medial pinnate setae; third segment with six pinnate setae decreasing in length toward outer margin and one longer naked spine followed by one minute, naked spine on lateral margin. Endopod 3-segmented; first segment with long pinnate seta distomedially and velum fringed with short setules; second segment with two long pinnate setae distomedially and velum fringed with short setules; third segment short, rounded, bearing six long pinnate setae decreasing in length toward outer margin and with few setules proximolaterally.

Leg III (Fig. 4C) biramous; sympod with large, pinnate seta medially, fringed with wide membrane along both margins, and ten sensilla scattered on medial surface. Exopod 3-segmented; first segment with slender distolateral serrate spine, distomedial pinnate seta and setules along medial margins; second segment with distolateral spine, distomedial pinnate seta and setules along inner and outer margins; third segment with three short distolateral spines, spine IV shorter than leg II, pinnate setae decreasing in length toward outer margin, and setules along inner and outer margins. Endopod 3-segmented; first segment with large rounded velum covering first two exopodal segments and most of velum inserted on second endopod segment, fringed with short setules; second segment with two long pinnate setae distomedially and small velum fringed with short setules; third segment bearing four long pinnate setae decreasing in length toward outer margin.

Leg IV (Fig. 4D) uniramous, brachiform; sympod robust, with short sensilla on inner surface. First segment with distolateral spinulate spine, pectinate membrane at base and spinules scattered along lateral margin; second segment with distolateral spine and pectinate membrane at insertion; third segment with one subapical and two apical spinulate spines, apical spine being almost twice as long as other two, one small spine and pectinate membrane at base of larger terminal spine. Leg V (Fig. 2D) located posterolaterally on ventral surface of genital complex, consisting of one short plumose seta and group of three short plumose setae on small papilliform process.

**Figure 5.**
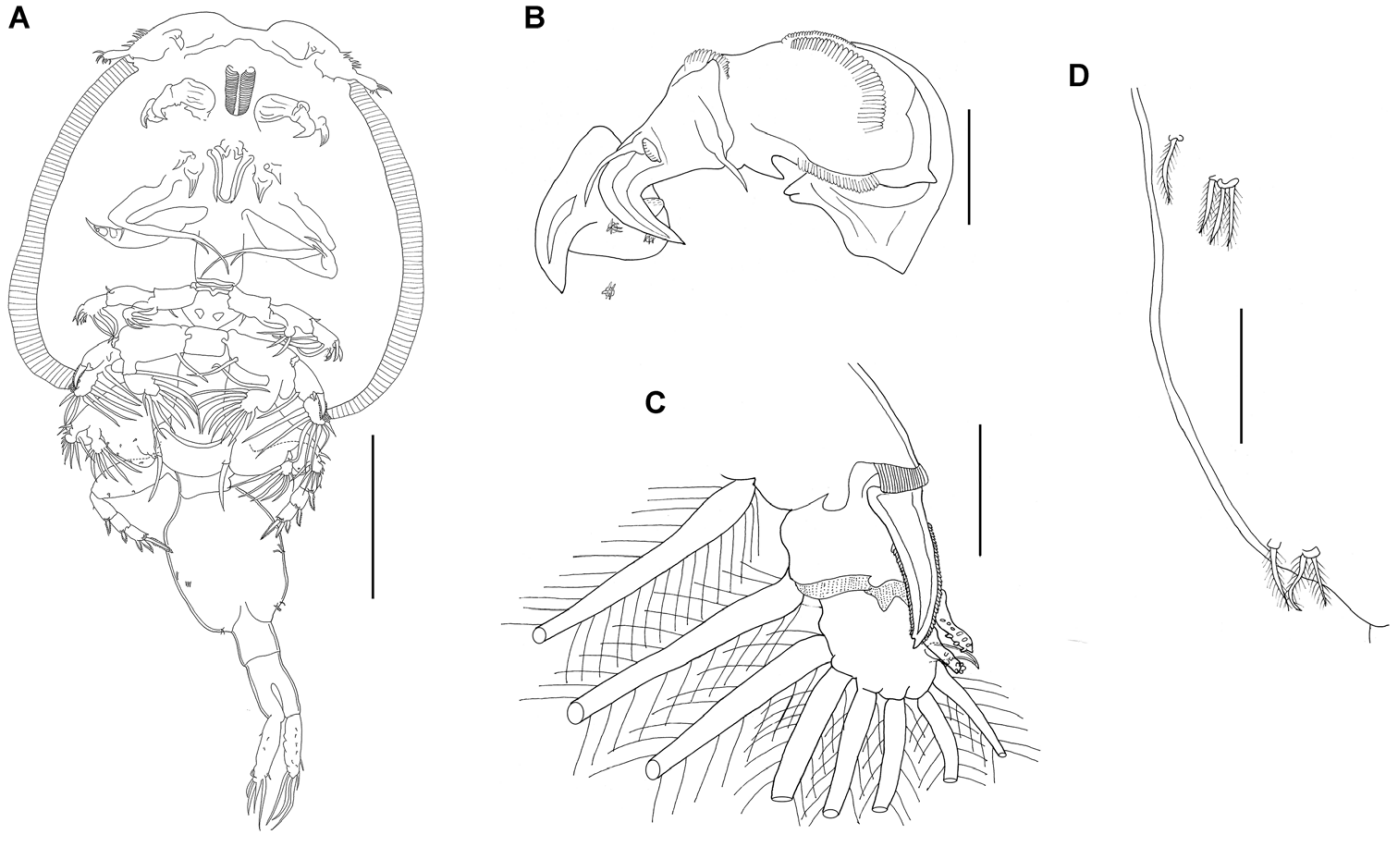
*Pupulinamantensis* sp. n., adult male. **A** habitus, ventral view **B** antenna and postantennal process **C** leg II **D** legs V and VI. Scale bars: **A**, 800 µm; **B–D**, 100 µm.

**Figure 6.**
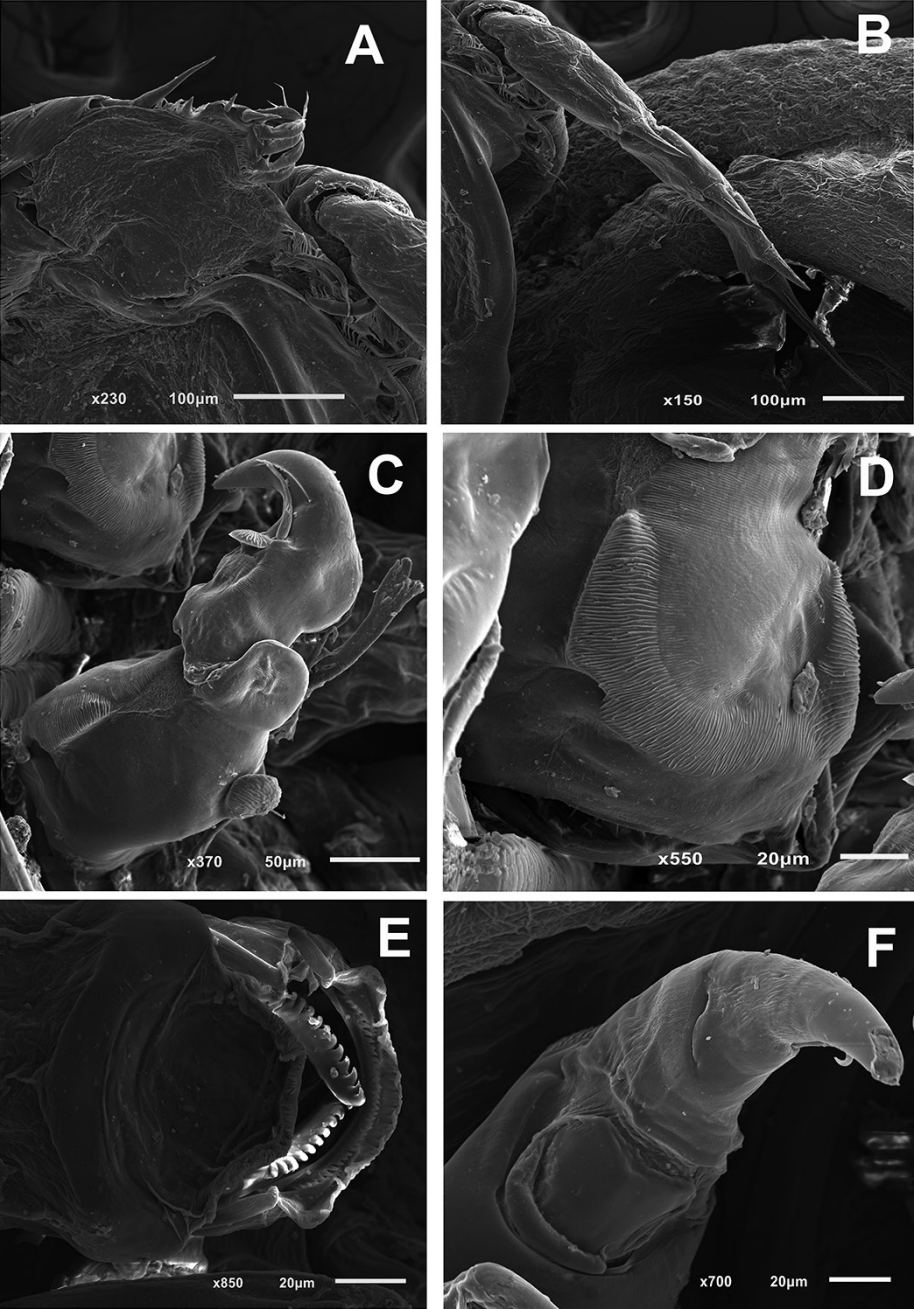
*Pupulinamantensis* sp. n., adult male. **A** leg III **B** leg IV **C** antenna and post-antennal process **D** details of second antennal segment showing adhesion pads **E** mouth tube and mandible **F** terminal segment of antenna showing adhesion pads.

*Male* (Figs 5–6). Body (Fig. 5A) 3.5–3.9 mm mm long (3.7 mm; n = 5) excluding caudal setae. Cephalothorax as in female but smaller (2.0 mm × 2.0 mm). Fourth pedigerous somite two times wider than long. Genital complex somewhat oval in outline. Abdomen 2-segmented with anal somite approximately two times longer than abdominal somite. Caudal rami slender, longer than wide; armed as in female. Body surface with small spinules similar to that in female.

Antennule as in female. Antenna (Figs 5B, 6A) different from that of female, 3-segmented; first segment unarmed; second segment with spine-like process in middle region and two corrugated adhesion pads in each margin of ventral surface (Fig. 6B); terminal segment sharply pointed, claw bearing proximal robust seta with small corrugated adhesion pad, slender seta in middle region and corrugated adhesion pads in the outer margin near base (Fig. 6C). Pair of larger corrugated adhesion pads located anteromedially to antennas (Fig. 6A). Postantennal process as in female. Mouth tube and mandible, similar to female (Fig. 6D). Maxillule similar to female, but only 2 setae in male. Maxilla and Maxilliped as in female.

Legs I, III, and IV as in female (Fig. 6E, F). Leg II as in female, except the second and third exopodal spine, both blunt spines bearing small spinules (Fig. 5C). Leg V (Fig. 5D) located on medial lateral margin of genital complex; consisting of one papilla with three plumose setae along one plumose seta slightly anterior to this group. Leg VI (Fig. 5D) located posterolaterally on ventral surface of genital complex; represented by two plumose setae arising from a single papilla, in addition to one plumose seta near base of papilla.

####### Remarks.

The specimens were identified as belonging to the genus *Pupulina* by their possession of the diagnostic characters described by Dojiri and Ho (2013) including the presence of posterolateral processes on the genital complex of the female, the presence of a small conical process posteromedial to the dentiform projection of the maxillule, the presence of a dentiform or membranous process immediately posterior to the maxilliped, a well-developed endopod of leg I, inflated outer margin of the first and second endopodal segments of leg II and first endopodal segment of leg III, distinctly 3-segmented rami of leg III, and the armature of the exopod of leg III.

The new species, *P.mantensis* sp. n., is mainly characterized by the rounded shape of the genital complex with slight posterolateral processes. Of the six valid species of the genus *Pupulina* worldwide, only *P.merira* have very short, rounded posterolateral processes. However, this species is easily separated from *P.mantensis* sp. n. by the possession of a squarish genital complex, less than half-length and width of cephalothorax (see fig. 4A in Dippenaar and Lebepe 2013), whereas the new species possesses a larger genital complex, rounded, around 3/4 length and 2/3 width of cephalothorax. *Pupulinacliffi* and *P.keiri* differs from *P.mantensis* sp. n. by the lack of posterolateral processes in the genital complex (see fig. 1A in Dippenaar and Lebepe 2013 and fig. 73C in Boxshall 2018, respectively). *Pupulinacliffi* also differs from *P.mantensis* sp. n. by the subquadrate shape of the genital complex with anterolateral corners slightly protruded (see fig. 1A in Dippenaar and Lebepe 2013). *Pupulinakeiri* also differs from *P.mantensis* sp. n. by the shape and proportion of the genital complex 1.2 times wider than long, with linear lateral margins and anterolateral corners slightly protruded (see fig. 73C in Boxshall 2018), although the author does not mention this last characteristic, whereas in *P.mantensis* sp. n. the genital complex is 1.2 times longer than wider, rounded and without anterolateral corners protruded. According to Wilson (1952) and Dojiri and Ho (2013), the members of *Pupulina* are clearly distinguished from other caligid genera by, among others features, the possession of posterolateral processes on the genital complex. However, *P.cliffi* and *P.keiri* are currently the only species of the genus without posterolateral processes (Dippenaar and Lebepe 2013). *Pupulinaflores*, *P.minor*, and *P.brevicauda* are easily separated from *P.mantensis* sp. n. by the possession of large posterolateral processes, extending approximately to midlength of abdomen in *P.flores* and *P.minor*, and beyond the caudal rami in *P.brevicauda*.

In addition to the characteristic mentioned above the new species differs from the other species by bearing sclerotized plate of the maxillule with dentiform process (without process dentiform each other except by *P.flores*), posterolateral process on genital complex not bulging (except by *Pupulinacliffi*, *P.merira*, and *P.keiri*), and sympod of leg IV with few spinules on surface (except by *Pupulinaminor* and *P.brevicauda*).

Among members of the genus *Pupulina*, males have been described for four species, i.e., *P.minor*, *P.brevicauda*, *P.flores*, and *P.keiri*. The male of *P.mantensis* sp. n. differs from other species by its possession of leg II with its second and third blunt exopodal spines bearing small spinules, and the presence of corrugated adhesion pads in each margin of ventral surface of the second antennal segment, a characters absent in its male congeners except in *P.keiri*. The male of a new species is more closely related to the male of *P.keiri*, which was described from the Mobulinae ray *Aetobatusocellatus* caught in Moreton Bay, Australia (Boxshall 2018). However, *P.mantensis* sp. n. is easily distinguished from *P.keiri* by the presence of small corrugated adhesion pads in the basis and the middle region of the terminal antennal segment, feature absent in *P.keiri* (see fig. 76D in Boxshall 2018). Furthermore, *P.mantensis* sp. n. male bears a couple of adhesion pads on the ventral anterior surface, an unusual structure hitherto undescribed in the genus.

*Pupulinamantensis* sp. n. female genital complex has slightly protruding posterolateral processes. It resembles that found in *P.merira* (see fig. 4A in Dippenaar and Lebepe 2013) and *P.cliffi* (see fig. 1A in Dippenaar and Lebepe 2013). According to Dippenaar and Lebepe (2013), species of this genus can be sorted by the absence, presence or shape of the posterolateral processes on the genital complex. The new species differs from its congeners by several characters, as follows *Pupulinamantensis* sp. n. has a sclerotized maxillulary plate with a dentiform process; the leg II third exopodal segment has two equally long spines; the abdomen represents almost 40% of genital complex; the sympod of leg IV bears a few spinules on ventral surface; second segment lacking distomedial seta or spine. Caudal rami are shorter than the abdomen in *P.mantensis* sp. n., thus diverging from *P.merira* (see fig. 4A–J in Dippenaar and Lebepe 2013).

In the new species, the female abdomen appears to have only one somite, which is an unusual feature in the genus. According to Dojiri and Ho (2013) and Wilson (1952) in the *Pupulina* genus, although the segmentation of the abdomen may be indistinct, it comprises 3-segments. Therefore, the number of segments as well as being a characteristic feature of the genus also can help in the identification of its species.

### Identification key to adult females of *Pupulina* species modified from Dippenaar and Lebepe (2013)

**Table:** 

1	Posterolateral processes on genital complex absent	**2**
–	Posterolateral processes on genital complex present	**3**
2	Abdomen as long as the genital complex and approximately 3.8 times longer than wide	*** P. cliffi ***
–	Abdomen less than half the length of the genital complex and approximately 2.3 times longer than wide	*** P. keiri ***
3	Posterolateral processes on genital complex long, extending beyond caudal rami	*** P. brevicauda ***
–	Posterolateral processes on genital complex not extending beyond caudal rami	**4**
4	Posterolateral processes on genital complex very short, rounded	**5**
–	Posterolateral processes on genital complex longer, reaching around mid-length of genital complex	**6**
5	Genital complex with a squarish shape, less than half length and width of cephalothorax; abdomen indistinctly 3-segmented; maxillule without dentiform process	*** P. merira ***
–	Genital complex rounded, large, around 3/4 length and 2/3 width of cephalothorax; abdomen in which more than one somite is indistinguishable; sclerotized plate of the maxillule with dentiform process	***P.mantensis* sp. n.**
6	Posterolateral processes with rounded tips; genital complex with a squarish shape and posterior border almost straight until abrupt change into posterolateral processes; abdomen almost same length as genital complex; caudal rami longer than abdomen; sclerotised plate lateral to maxillulary palp, small, not extending to bulging area of praecoxal endite with posteriorly rounded protrusion	*** P. minor ***
–	Posterolateral processes with pointed tips; genital complex with more rounded shape and posterior border gradually extending into posterolateral processes; abdomen longer than genital complex; sclerotised plate lateral to maxillulary palp, long, sharply pointed, reaching beyond bulging area of precoxal endite	*** P. flores ***

## Discussion

We found *Pupulinamantensis* sp. n. parasitizing six individuals of the white-spotted eagle ray A.cf.narinari, belonging to the elasmobranch family Myliobatidae Bonaparte, 1835; subfamily Myliobatinae Bonaparte, 1835. According to Wilson (1952), Dojiri and Ho (2013), and Dippenaar and Lebepe (2013), members of the genus *Pupulina* appear to be specific for species of rays of the family Mobulidae. However, the family Myliobatidae that contains the subfamilies Mobulinae Gill, 1893, Myliobatinae and Rhinopterinae Jordan & Evermann, 1896 (Bailly 2015) replaced the family Mobulidae (currently uncertain). Five of the six valid species of the genus *Pupulina* (*Pupulinaflores* van Beneden, 1892; *P.brevicauda* Wilson, 1952; *P.minor* Wilson, 1952; *P.cliffi* Dippenaar & Lebepe, 2013; *P.merira* Dippenaar & Lebepe, 2013) have been recorded as parasites of the rays *Mobulalucasana* (Beebe and Tee-Van 1938) [= *Mobulathurstoni* (Lloyd, 1908)], *Mobuladiabolus* (Shaw, 1804) [= *Mobulamobular* (Bonnaterre, 1788)], *M.kuhlii* (Müller & Henle, 1841), *M.eregoodootenkee* (Bleeker, 1859) and *Mantabirostris* (Walbaum, 1792), all belonging to the subfamily Mobulinae. This finding of *P.mantensis* sp. n. as a parasite on the myliobatine ray A.cf.narinari, confirm the host range (new subfamily) expansion among elasmobranchs described recently for *P.keiri* from *Aetobatusocellatus* Kuhl, 1823 (Boxshall 2018).

As stated above, we distinguished two morphotypes of A.cf.narinari based on its dorsal spot pattern. *Pupulinamantensis* sp. n. was found in the ray morphotype 1 only (fully white dorsal spots). *Aetobatusnarinari* has been characterized by showing morphological differences related to distinct geographic regions (Compagno et al. 2005, Kyne et al. 2006); moreover, molecular data suggest that *A.narinari* is a species complex with at least two distinct species and probably also two subspecies (Richards et al. 2009). This species complex of *A.narinari* could show a distinctive parasitic fauna among morphotypes. Marie and Justine (2005) first argued that *A.narinari* could be a species complex because of differences in the diversity of parasitic monogenean helminths occurring in populations of *A.narinari* from different geographic regions. The sample size is too small to speculate and advance *P.mantensis* sp. n. as a potential specific parasite for this morphotype. However, we provide the information about each morphotype to make it available when the taxonomic status of *A.narinari* is properly solved.

*Pupulinamantensis* sp. n. represents the third record of parasitic copepods from *A.narinari* and the second record of the genus *Pupulina* from Ecuador (Wilson 1935, Pollerspöck and Straube 2015). Currently, the metazoan parasite fauna of *Aetobatusnarinari* comprises 56 species of different groups: (Cestoda (36 sp.); Monogenea (7 sp.), Nematoda (6 sp.), Isopoda (4 sp.), and Hirudinea (1 sp.)) (Pollerspöck and Straube 2015). Only two copepods species, *Eudactylinahombosteli* Deets, 1994 (Dippenaar and Jordaan 2007) and *Euryphorussuarezi* (Morales-Serna, Rodríguez-Santiago and Gómez 2016), have been reported from *A.narinari*. The first record of *Pupulina* from Ecuador was *P.flores* van Beneden, 1892, from the giant ray *Mantabirostris* from Galápagos Islands (Wilson 1935), but the genus may well be represented by other species in this geographical region because of the abundance and diversity of potential hosts belonging to the subfamilies Mobulinae and Myliobatinae (Denkinger and Vinueza 2014).

## Supplementary Material

XML Treatment for Pupulina mantensis
